# Anti-Inflammatory Potential of Essential Oil from the Heart-Wood of the Folk Medicinal Tree *Cinnamomum kanehirai* Hayata in Macrophages

**DOI:** 10.3390/ijms26115419

**Published:** 2025-06-05

**Authors:** May-Lan Liu, Pang-Yen Liu, Louis Kuoping Chao, Tzu-Jung Yang, Lan-Hui Li, Yih-Ming Weng, Sarana Rose Sommano, Yuwalee Unpaprom, Rameshprabu Ramaraj, Chen-Lung Ho, Kuo-Feng Hua

**Affiliations:** 1 Department of Food Science, National Chiayi University, Chiayi 600355, Taiwan; m0931798072@gmail.com (M.-L.L.); ymweng@mail.ncyu.edu.tw (Y.-M.W.); 2 Department of Early Childhood Educare, WuFeng University, Chiayi 621303, Taiwan; 3 Division of Cardiology, Department of Internal Medicine, Tri-Service General Hospital, National Defense Medical Center, Taipei 114202, Taiwan; liupy@mail.ndmctsgh.edu.tw; 4 Department of Cosmeceutics, China Medical University, Taichung 406040, Taiwan; kuoping@mail.cmu.edu.tw; 5 Division of Wood Cellulose, Taiwan Forestry Research Institute, Taipei 10066, Taiwan; yangbe313@gmail.com; 6 Department of Laboratory Medicine, Linsen Chinese Medicine and Kunming Branch, Taipei City Hospital, Taipei 108203, Taiwan; A1525@tpech.gov.tw; 7 Plant Bioactive Compound Laboratory (BAC), Faculty of Agriculture, Chiang Mai University, Chiang Mai 50200, Thailand; sarana.s@cmu.ac.th; 8 Center of Excellence in Agro Bio-Circular-Green Industry (Agro BCG), Faculty of Agro-Industry, Chiang Mai University, Chiang Mai 50100, Thailand; 9 Department of Plant and Soil Sciences, Faculty of Agriculture, Chiang Mai University, Chiang Mai 50200, Thailand; 10 Sustainable Resources and Sustainable Engineering Research Lab, Maejo University, Chiang Mai 50290, Thailand; yuwaleeun@gmail.com (Y.U.); rrameshprabu@gmail.com (R.R.); 11 Program in Biotechnology, Faculty of Science, Maejo University, Chiang Mai 50290, Thailand; 12 School of Renewable Energy, Maejo University, Chiang Mai 50290, Thailand; 13 Department of Biotechnology and Animal Science, National Ilan University, Ilan 260007, Taiwan; 14 Department of Medical Research, China Medical University Hospital, China Medical University, Taichung 404328, Taiwan

**Keywords:** anti-inflammatory agent, NLRP3 inflammasome, *Cinnamomum kanehirai* Hayata, essential oils, cytokines, macrophages

## Abstract

Inflammation is a vital physiological response that plays a crucial role in regulating host defense against pathogens while maintaining tissue homeostasis. Inflammasomes, a family of protein complexes, are responsible for controlling the expression of pro-inflammatory cytokines IL-1β and IL-18, and they play significant roles in inflammatory responses. However, dysregulated inflammation can become a risk factor for the pathogenesis of various diseases. The discovery of anti-inflammatory substances derived from natural products represents an important strategy for new drug development. In this study, we found that the essential oil derived from the heartwood of *Cinnamomum kanehirai* Hayata (EOC) exhibits anti-inflammatory activities by inhibiting the NLRP3, NLRP1, NLRC4, AIM2, and non-canonical inflammasomes in macrophages. EOC also suppresses the expression of NLRP3, TNF-α, IL-6, and NO in LPS-activated macrophages. The mechanisms underlying the anti-inflammatory activity of EOC were shown to involve a reduction in reactive oxygen species production and NF-κB activation. Furthermore, terpinen-4-ol may be the key anti-inflammatory compound present in EOC. These results suggest that EOC has potential as an anti-inflammatory agent for future development.

## 1. Introduction

Inflammation is a crucial cellular process involved in host defense against microbial infections and in wound healing [1,2]. However, uncontrolled inflammation can damage tissues and organs, leading to the development and progression of various diseases [3,4]. While inflammation is typically triggered by infectious agents, increasing evidence suggests that it can also be induced by non-infectious factors, such as aging and obesity, which contribute to “inflammaging” and “metainflammation”, respectively [5,6]. The NLRP3 inflammasome is a protein complex composed of NLR family pyrin domain containing 3 (NLRP3), apoptosis-associated speck-like protein (ASC), and caspase-1, and it regulates the maturation and secretion of pro-inflammatory cytokines interleukin (IL)-1β and IL-18 [7]. In both inflammaging and metainflammation, the NLRP3 inflammasome is activated and plays significant roles in these conditions [8]. Activation of the NLRP3 inflammasome can be triggered by metabolic danger signals, such as cholesterol crystals (related to atherosclerosis), uric acid crystals (associated with gout), fatty acids (linked to diabetes), and aging-related factors like aggregated and misfolded proteins, including fibrillar amyloid-β (in Alzheimer’s disease) and α-synuclein (in Parkinson’s disease) [9]. Targeting the NLRP3 inflammasome presents a promising strategy for disease prevention and therapeutic intervention [10].

Full activation of the NLRP3 inflammasome requires two signals: a priming signal and an activation signal. The priming signal generally arises from toll-like receptors and cytokine receptors, which induce the production of reactive oxygen species (ROS) and activate NF-κB. This cascade leads to the transcriptional induction of NLRP3 and the precursor of IL-1β (proIL-1β) [11]. The activation signals originate from disease-associated stimuli that promote the assembly of the NLRP3 inflammasome and the activation of caspase-1, ultimately resulting in the maturation and secretion of IL-1β and IL-18 [9]. It has been demonstrated that mitochondrial damage, characterized by mitochondrial ROS production and loss of mitochondrial membrane integrity, serves as a significant activation signal for the NLRP3 inflammasome [12]. Natural products possess diverse chemical structures, which contribute to their multiple biological functions. Increasing data support the notion that natural products can serve as novel, practical, and accessible agents for the treatment of inflammatory diseases by targeting the NLRP3 inflammasome [13,14].

*Cinnamomum kanehirai* Hayata, commonly known as the Taiwan camphor tree, is an evergreen species belonging to the Lauraceae family and is native to Taiwan. The wood of *C. kanehirai* Hayata is highly valued for its toughness and durability, making it suitable for furniture and construction. Additionally, this tree serves as the primary host for the medicinal fungus *Antrodia cinnamomea*. The leaves of *C. kanehirai* Hayata are used to make tea, which is believed to promote health. Research indicates that *C. kanehirai* Hayata contains active compounds with potential biomedical applications. For example, ethanol extracts from its leaves demonstrate antioxidant activity and induce caspase-3-dependent apoptosis in human hepatoma cells [15,16]. Furthermore, essential oils extracted from the twigs and leaves of *C. kanehirai* Hayata exhibit antibacterial properties [17]. However, the effects of essential oils from the heartwood of *C. kanehirai* Hayata on the inflammatory response, particularly regarding the NLRP3 inflammasome, remain unexplored.

In this study, we investigate the inhibitory potential of essential oil isolated from the heartwood of *C. kanehirai* Hayata (EOC) against the NLRP3 inflammasome and its effects on the expression of inflammatory mediators. We use macrophages as a cellular model to assess the impact of EOC on NLRP3 inflammasome activation and to elucidate the underlying mechanisms responsible for its anti-inflammatory activity.

## 2. Results

### 2.1. EOC Inhibits the NLRP3 Inflammasome Activated by Adenosine Triphosphate (ATP)

**Figure 1:**
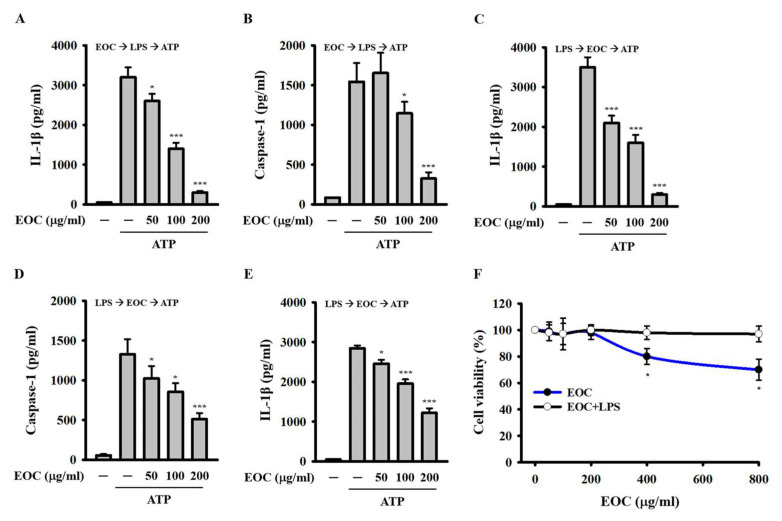
Effect of EOC on NLRP3 inflammasome activation induced by ATP. (**A**,**B**) J774A.1 macrophages were preincubated with EOC for 0.5 h, followed by treatment with 1 μg/mL LPS and 5 mM ATP. The expression of IL-1β (**A**) and active caspase-1 (**B**) in the supernatants was analyzed using ELISA. (**C**,**D**) 1 μg/mL LPS-primed J774A.1 macrophages were stimulated with 5 mM ATP. The expression of IL-1β (**C**) and active caspase-1 (**D**) in the supernatants was analyzed using ELISA. (**E**) 1 μg/mL LPS-primed THP-1 macrophages were activated with 5 mM ATP. The supernatant IL-1β levels were quantified using ELISA. (**F**) J774A.1 macrophages were incubated with EOC, with or without 1 μg/mL LPS, for 24 h. Cell viability was evaluated using the Alamar Blue assay. ELISA and cell viability results are presented as mean ± SD from three independent experiments. Statistical significance is indicated as * *p* < 0.05 and *** *p* < 0.001 compared to ATP-activated (**A**–**E**) or control cells (**F**).

To investigate whether EOC exerts inhibitory activity on the NLRP3 inflammasome, J774A.1 macrophages were pretreated with EOC for 0.5 h, followed by stimulation with 1 μg/mL lipopolysaccharide (LPS) for 5.5 h and subsequent activation with 5 mM ATP for an additional 0.5 h. The concentration of IL-1β in the supernatants was measured using enzyme-linked immunosorbent assay (ELISA). We found that EOC reduced IL-1β production in a dose-dependent manner (Figure 1A). Under the same conditions, EOC also decreased the concentration of caspase-1 in the supernatants (Figure 1B). These results indicate that EOC inhibits NLRP3 inflammasome activation in macrophages. To further assess whether EOC targets the inflammasome activation signals, J774A.1 macrophages were first primed with 1 μg/mL LPS for 5.5 h, followed by treatment with EOC for 0.5 h, and then activated with 5 mM ATP for another 0.5 h. ELISA analysis showed that EOC continued to reduce IL-1β (Figure 1C) and caspase-1 (Figure 1D) levels in a dose-dependent manner. To determine whether EOC’s inhibitory effect on IL-1β expression is restricted to murine macrophages, 1 μg/mL LPS-primed human THP-1 macrophages were treated with EOC for 0.5 h and subsequently activated with 5 mM ATP for an additional 0.5 h. Consistent with the results in J774A.1 cells, EOC significantly reduced IL-1β production in a dose-dependent manner in THP-1 cells (Figure 1E). To rule out the possibility that the observed reductions in IL-1β and caspase-1 were due to cytotoxicity, J774A.1 macrophages were incubated with various concentrations of EOC for 24 h, with or without 1 μg/mL LPS, and cell viability was assessed using the Alamar Blue assay. EOC alone did not significantly affect cell viability at concentrations up to 200 µg/mL but exhibited cytotoxic effects at 400 and 800 µg/mL. Interestingly, in the presence of LPS, EOC did not affect cell viability even at concentrations up to 800 µg/mL (Figure 1F). Collectively, these findings suggest that EOC inhibits NLRP3 inflammasome activation, at least in part, by modulating ATP-induced activation signals.

### 2.2. EOC Inhibits the NLRP3 Inflammasome Activated by Multiple NLRP3 Activators

**Figure 2:**
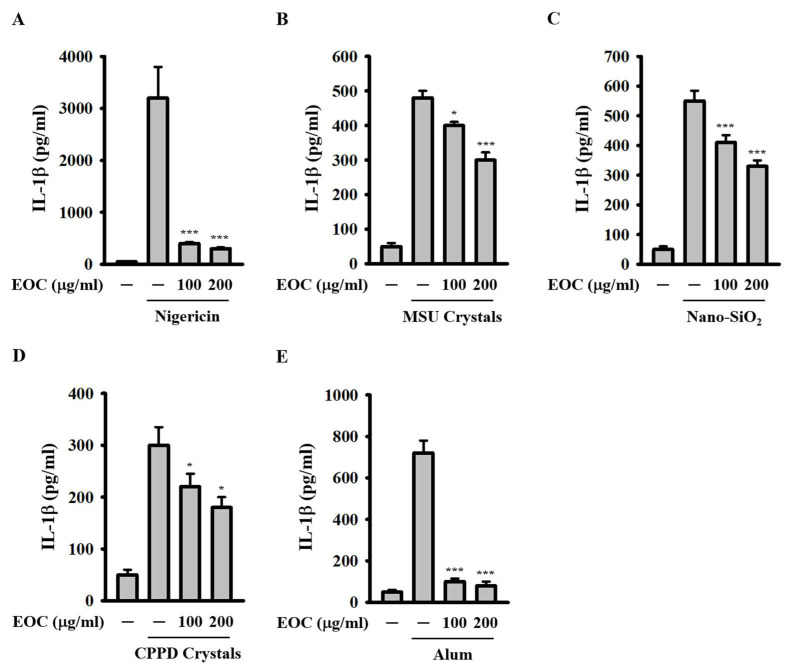
Effect of EOC on IL-1β expression induced by multiple NLRP3 activators. 1 μg/mL LPS-primed J774A.1 macrophages were incubated with EOC for 0.5 h, followed by activation with 10 μM nigericin (**A**), 100 μg/mL MSU crystals (**B**), 100 μg/mL Nano-SiO_2_ (**C**), 10 μg/mL CPPD crystals (**D**), and 100 μg/mL Alum (**E**). The levels of IL-1β in the supernatants were quantified using ELISA. Results are presented as mean ± SD from three independent experiments. Statistical significance is marked as * *p* < 0.05 and *** *p* < 0.001 compared to cells activated by NLRP3 inflammasome.

To confirm the inhibitory activity of EOC on the NLRP3 inflammasome, we tested its effect on IL-1β expression in macrophages activated by various NLRP3 activators, other than ATP. 1 μg/mL LPS-primed J774A.1 macrophages were incubated with EOC for 0.5 h, followed by activation with 10 μM nigericin for 0.5 h, or with 100 μg/mL monosodium urate (MSU) crystals, 100 μg/mL SiO_2_ nanoparticles (Nano-SiO_2_), 10 μg/mL calcium pyrophosphate dihydrate (CPPD) crystals, and 100 μg/mL aluminum hydroxide (Alum) for 24 h. Using ELISA, we found that EOC at concentrations of 100 or 200 μg/mL significantly decreased IL-1β expression in macrophages activated by nigericin (Figure 2A), MSU crystals (Figure 2B), Nano-SiO_2_ (Figure 2C), CPPD crystals (Figure 2D), and Alum (Figure 2E). These results indicate that EOC inhibits the NLRP3 inflammasome activated by multiple NLRP3 activators.

### 2.3. EOC Inhibits Inflammasomes Beyond the NLRP3 Inflammasome

**Figure 3:**
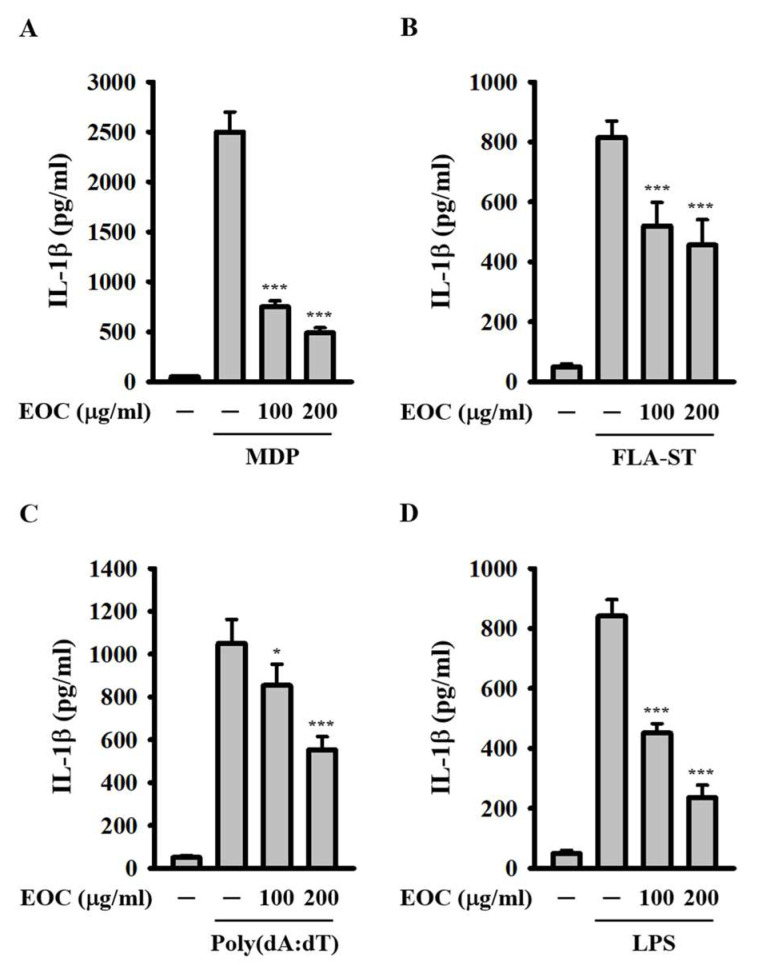
Effect of EOC on IL-1β expression induced by multiple NLRP3 inflammasome activators. 1 µg/mL LPS-primed (**A**–**C**) or 1 µg/mL Pam3CSK4-primed (**D**) J774A.1 macrophages were incubated with EOC for 0.5 h, followed by transfection with 10 µg/mL MDP (**A**), 1 µg/mL FLA-ST (**B**), 2 µg/mL Poly(dA:dT) (**C**), and 2 µg/mL LPS (**D**). The levels of IL-1β in the supernatants were measured using ELISA. Results are presented as mean ± SD from three independent experiments. Statistical significance is indicated as * *p* < 0.05 and *** *p* < 0.001 compared to cells activated by the inflammasome.

To determine whether EOC specifically inhibits the NLRP3 inflammasome or affects other inflammasomes as well, we incubated 100 μg/mL LPS-primed J774A.1 macrophages with EOC for 0.5 h. This was followed by the transfection of 10 µg/mL muramyl dipeptide (MDP), 1 µg/mL flagellin from *Salmonella typhimurium* (FLA-ST), and 2 µg/mL Poly(dA:dT) for 6 h, which serve as inducers for the NLRP1, NLRC4, and AIM2 inflammasomes, respectively. Using ELISA, we found that EOC significantly reduced IL-1β expression induced by the transfection of MDP (Figure 3A), FLA-ST (Figure 3B), and Poly(dA:dT) (Figure 3C), indicating that EOC inhibits the activation of the NLRP1, NLRC4, and AIM2 inflammasomes. Additionally, we investigated whether EOC affects non-canonical inflammasome activation. J774A.1 macrophages were primed with 1 µg/mL synthetic triacylated lipopeptide (Pam3CSK4) for 5.5 h and then incubated with EOC for 0.5 h before activation via 2 µg/mL LPS transfection for 6 h. ELISA results showed that EOC significantly reduced IL-1β expression induced by LPS transfection (Figure 3D). These findings indicate that EOC is not a specific inhibitor of the NLRP3 inflammasome; rather, it also inhibits the NLRP1, NLRC4, AIM2, and non-canonical inflammasomes in macrophages.

### 2.4. EOC Inhibits the Priming Signals of the NLRP3 Inflammasome

**Figure 4:**
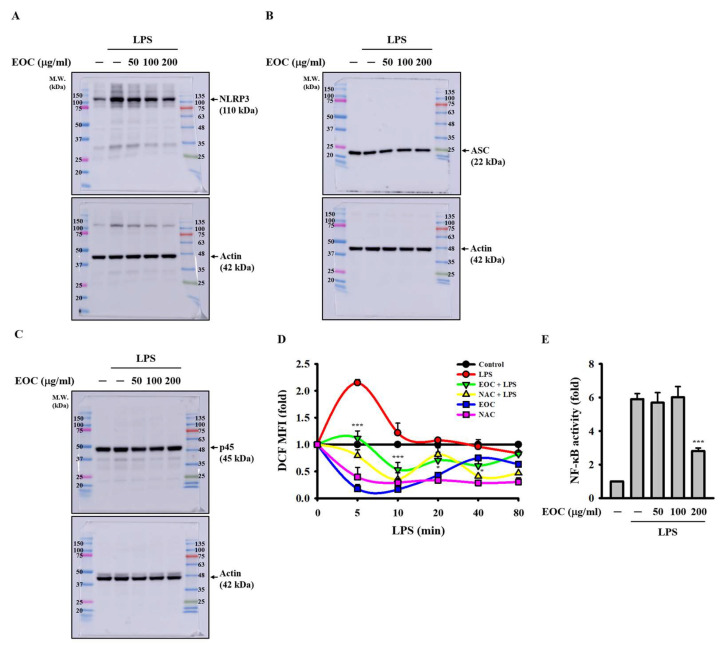
The effect of EOC on priming signals of the NLRP3 inflammasome. (**A**–**C**) J774A.1 macrophages were treated with EOC for 0.5 h, followed by 1 μg/mL LPS activation for 6 h. The protein expression of NLRP3 (**A**), ASC (**B**), and caspase-1 (p45) (**C**) were measured using Western blot. (**D**) J774A.1 macrophages were treated with 100 μg/mL EOC for 0.5 h, followed by 1 μg/mL LPS activation for 5 to 80 min. The cells were stained with 2 μM CM-H_2_DCFDA and analyzed using a spectrophotometer. (**E**) NF-κB reporter cells were incubated with EOC for 0.5 h and then activated with 1 μg/mL LPS for 24 h. The NF-κB transcriptional activity was measured using QUANTI-Blue solution. ROS and NF-κB results are presented as mean ± SD from three independent experiments. Statistical significance is indicated as * *p* < 0.05 and *** *p* < 0.001 compared to LPS-activated cells.

To elucidate the molecular mechanism by which EOC inhibits NLRP3 inflammasome activation, we investigated whether EOC interferes with the priming signals required for inflammasome activation. First, we examined the effect of EOC on the expression of NLRP3 in LPS-activated macrophages. J774A.1 macrophages were pretreated with EOC for 0.5 h and then stimulated with LPS for 6 h. Western blot analysis showed that EOC dose-dependently reduced the levels of NLRP3 (Figure 4A). In contrast, the expression of ASC (Figure 4B) and caspase-1 (p45) (Figure 4C)—both constitutively expressed in macrophages—remained unaffected by EOC treatment. Next, we explored whether EOC modulates ROS production, a key upstream event in NLRP3 inflammasome priming. J774A.1 macrophages were pretreated with 100 µg/mL EOC for 0.5 h and then stimulated with 1 µg/mL LPS for 5–80 min. Intracellular ROS levels were assessed using 2 µM CM-H_2_DCFDA staining followed by fluorescence analysis. EOC significantly suppressed LPS-induced ROS production (Figure 4D). We also evaluated the effect of EOC on NF-κB transcriptional activity, another critical event in the priming phase. J-Blue cells, an NF-κB reporter cell line, were pretreated with EOC for 0.5 h and subsequently stimulated with 1 µg/mL LPS for 24 h. EOC markedly reduced NF-κB transcriptional activity (Figure 4E). Collectively, these findings suggest that EOC inhibits NLRP3 inflammasome activation, at least in part, by attenuating LPS-induced priming signals through suppression of both ROS production and NF-κB activation.

### 2.5. EOC Enhances Mitochondrial Damage

**Figure 5:**
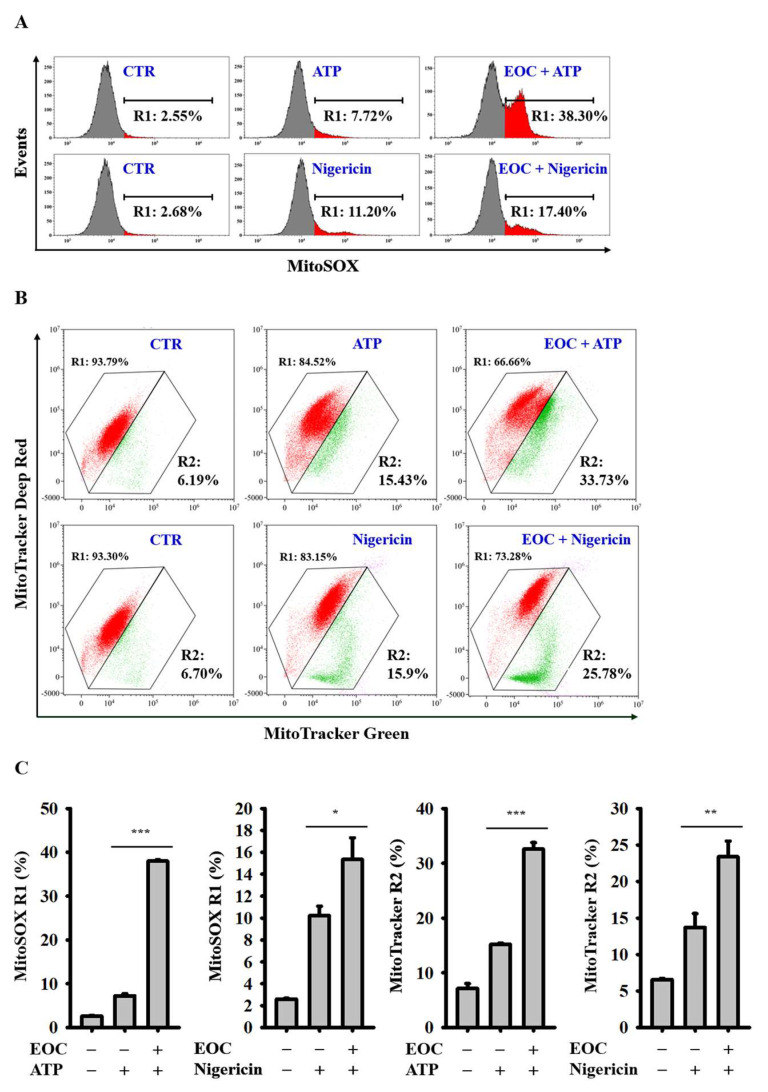
The effect of EOC on the mitochondrial damage. 1 μg/mL LPS-primed J774A.1 macrophages were incubated with 100 μg/mL EOC for 0.5 h and then activated with 5 mM ATP or 10 μM nigericin for an additional 0.5 h. The cells were stained with 5 nM MitoSOX (**A**) or with a combination of 25 nM MitoTracker Deep Red and 25 nM MitoTracker Green (**B**) for 15 min. The fluorescence signals were then analyzed using flow cytometry. The flow cytometry images represent individual experiments, while the histogram provides quantification expressed as the mean ± SD for these three experiments (**C**). Statistical significance is indicated as * *p* < 0.05, ** *p* < 0.01 and *** *p* < 0.001.

To determine whether EOC inhibits the NLRP3 inflammasome by suppressing activation signals, we assessed its effect on mitochondrial damage-a key event leading to NLRP3 inflammasome activation. 1 μg/mL LPS-primed J774A.1 macrophages were treated with 100 μg/mL EOC for 0.5 h, followed by stimulation with 5 mM ATP or 10 μM nigericin for an additional 0.5 h. The cells were then stained with 5 nM MitoSOX and 25 nM MitoTracker, fluorescent probes specific for mitochondrial ROS and mitochondrial membrane integrity, respectively. The fluorescent signals were analyzed via flow cytometry. Our results showed that EOC did not reduce but significantly increased mitochondrial ROS production (Figure 5A,C). Furthermore, EOC also intensified the loss of mitochondrial membrane integrity (Figure 5B,C). These findings suggest that EOC does not inhibit the NLRP3 inflammasome by mitigating mitochondrial damage.

### 2.6. EOC Inhibits the Inflammatory Response Independent of the NLRP3 Inflammasome

**Figure 6:**
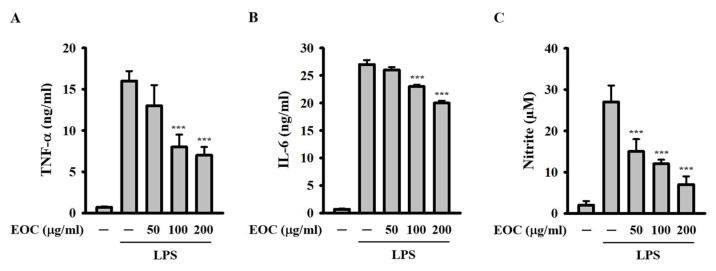
Effect of EOC on the inflammatory response independent of the NLRP3 inflammasome. J774A.1 macrophages were incubated with EOC for 0.5 h and then activated with 1 μg/mL LPS for 24 h. The expression levels of TNF-α (**A**) and IL-6 (**B**) in the supernatants were assessed using ELISA, while NO production in the supernatants was measured using the Griess reaction (**C**). The results from the ELISA and Griess reaction are presented as mean ± SD from three independent experiments. Statistical significance is indicated as *** *p* < 0.001 compared to the LPS-activated cells.

To explore the anti-inflammatory activity of EOC, we tested its effect on LPS-induced inflammatory responses that are independent of the NLRP3 inflammasome. J774A.1 macrophages were incubated with EOC for 0.5 h, followed by activation with 1 μg/mL LPS for 24 h. ELISA results indicated that EOC significantly reduced the expression of tumor necrosis factor-α (TNF-α) (Figure 6A) and IL-6 (Figure 6B). Additionally, the Griess reaction revealed that EOC decreased nitric oxide (NO) production in a dose-dependent manner (Figure 6C). These results suggest that EOC not only inhibits inflammasome activation but also reduces the expression of inflammatory mediators, independently of inflammasome pathways.

### 2.7. Effect of Major Compounds of EOC on NO Production

**Figure 7:**
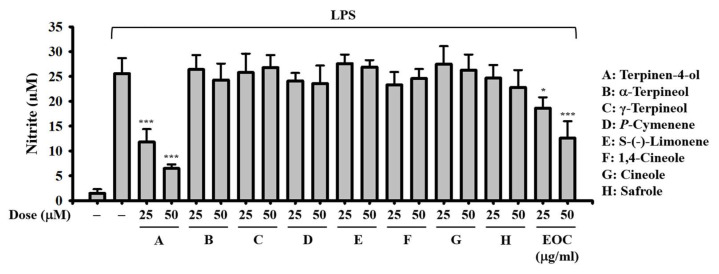
Effect of major compounds of EOC on NO production. 774A.1 macrophages were incubated with 25 and 50 μM test compounds or 25 and 50 μg/mL EOC for 0.5 h and then activated with 1 μg/mL LPS for 24 h. The NO production in the supernatants was measured using the Griess reaction. The results are presented as mean ± SD from three independent experiments. Statistical significance is indicated as * *p* < 0.05 and *** *p* < 0.001 compared to the LPS-activated cells.

Using gas chromatography–mass spectrometry (GC–MS) analysis, we identified 23 components in EOC, with the primary components being Safrole (34.5%), Terpinen-4-ol (20.5%), 1,4-Cineole (11.9%), *γ*-Terpinene (9.7%), *p*-Cymene (9.3%), and Limonene (3.3%). The predominant categories of these compounds were oxygenated monoterpenes (72.6%) and monoterpene hydrocarbons (25.4%) (Table 1). NO production by LPS-activated macrophages is widely recognized as a representative indicator of cellular inflammation. Upon stimulation with LPS, macrophages upregulate inducible nitric oxide synthase, resulting in the overproduction of NO, a key pro-inflammatory mediator involved in the regulation of cardiovascular function, metabolism, neurotransmission, and immune responses [18]. According to a recent systematic review and meta-analysis, the measurement of NO levels in LPS-stimulated RAW264.7 macrophages is a widely adopted and reliable method for evaluating the anti-inflammatory potential of various compounds, including plant-derived extracts and essential oils [19]. Therefore, in our study, NO production was assessed as a primary indicator to evaluate the anti-inflammatory activity of EOC. To determine the major bioactive components responsible for the anti-inflammatory activity of EOC, we evaluated the effects of commercially available key compounds on NO production in LPS-activated macrophages. J774A.1 macrophages were incubated with the test compounds for 0.5 h, followed by a 24 h activation with 1 μg/mL LPS. The NO concentration in the supernatants was analyzed using the Griess reaction. Our findings indicated that terpinen-4-ol at concentrations of 25 and 50 µM significantly inhibited NO production, while other test compounds did not affect NO levels, suggesting that terpinen-4-ol may be the key contributor to the NO inhibitory activity of EOC (Figure 7).

## 3. Discussion

The NLRP3 inflammasome responds to both microbial infections and sterile stimuli, leading to the expression of IL-1β and IL-18 and resulting in inflammation [7,9]. In recent years, the coronavirus disease 2019 (COVID-19) has significantly impacted public health and the global economy [23]. The NLRP3 inflammasome is activated in response to severe acute respiratory syndrome coronavirus 2 (SARS-CoV-2) infection, and its activation status is positively correlated with disease severity and mortality in patients [24]. Inhibiting the NLRP3 inflammasome may reduce hyperinflammation and lung injury in COVID-19 patients, as well as in experimental mouse models [25,26,27]. In this study, we demonstrated that EOC inhibits the NLRP3 inflammasome, suggesting that EOC may have beneficial effects on COVID-19. However, further investigations are necessary to validate this hypothesis. The NLRP3 inflammasome plays significant roles in aging and metabolism-related disorders, as it can be activated by MSU crystals and CPPD crystals, which are the etiological agents of the acute inflammatory conditions gout and pseudogout, respectively [28,29]. We found that EOC significantly reduced IL-1β expression in macrophages activated by both MSU and CPPD crystals, suggesting that EOC may help alleviate gouty inflammation. Additionally, EOC also decreased IL-1β expression induced by nano-SiO_2_, indicating that EOC may improve lung inflammation related to air pollution, such as PM_2_._5_ exposure [30].

The AIM2 inflammasome is a cytoplasmic sensor that recognizes intracellular double-stranded DNA derived from bacteria and DNA viruses [31]. We found that EOC inhibits IL-1β expression induced by intracellular poly(dA/dT), indicating that EOC may reduce inflammation caused by microbial infections. Interestingly, one study observed that larger quantities of double-stranded DNA colocalized with AIM2 in the aortic arch of mice in advanced stages of atherosclerosis. AIM2-deficient mice exhibited reduced IL-1β and IL-18 expression within atherosclerotic lesions and improved histopathological features associated with lesion stability [32]. These findings suggest that EOC may also have a cardiovascular protective effect by inhibiting the AIM2 inflammasome. Additionally, EOC may reduce inflammation caused by the Gram-negative bacterium *S. typhimurium* infection, as it inhibits IL-1β expression in macrophages induced by flagellin isolated from *S. typhimurium* [33].

We found that EOC not only inhibits the NLRP3 inflammasome activated by various stimuli but also suppresses other inflammasomes beyond NLRP3. The diverse biological functions of EOC may arise from its wide array of chemical constituents. Generally, plant essential oils contain dozens of components in varying concentrations, and in some instances, they may consist of hundreds of different compounds [34]. In this study, we identified 23 components in EOC, with 8 compounds present at concentrations exceeding 1%. We observed that terpinen-4-ol, one of the major compounds in EOC (20.53%), significantly reduced NO production in LPS-activated J774A.1 macrophages. Previously, we reported that terpinen-4-ol, the principal compound in the essential oil derived from *Liquidambar formosana* leaves, also decreased NO production in LPS-activated J774A.1 macrophages [35]. Notably, terpinen-4-ol has been shown to reduce colonic NLRP3 inflammasome activation in dextran sulfate sodium (DSS)-treated mice and to diminish NLRP3 inflammasome activation in LPS-activated RAW264.7 macrophages [36]. However, this finding necessitates further verification, as RAW264.7 macrophages may theoretically lack NLRP3 inflammasome activation due to their genetic deficiency in ASC [37]. Additionally, while our study found that alpha-terpineol (0.95% in EOC) did not significantly reduce NO production in LPS-activated J774A.1 macrophages, it did significantly decrease NO production in LPS-activated mouse peritoneal macrophages [38]. The apparent discrepancy between our current study and previous research may be attributed to the differences in cell types employed. It is important to note that in the study conducted by Oliveira et al. (2012) [38], the NO inhibitory activity of alpha-terpineol at a concentration of 1 µg/mL was comparable to that at 100 µg/mL, indicating a lack of a dose-response effect. Furthermore, 1,8-Cineole, a minor compound in EOC (0.53%), has been reported to inhibit the activity of HSP90, thereby suppressing the NLRP3 inflammasome in macrophages and alleviating DSS-induced intestinal barrier damage in mice [39]. 1,8-Cineole also increases SIRT2 expression and inhibits the deacetylation of NLRP3, which leads to reduced pyroptosis in human umbilical vein endothelial cells [40]. Further investigation is needed to identify the specific compounds responsible for the inhibition of inflammasomes.

Since EOC is not a homogeneous solution composed of a single compound, the diversity of its components significantly influences its chemotype and, consequently, its bioactivity. One limitation of this study is the quality control of the EOC used. Several factors can impact the chemical composition of plant essential oils, including the growth conditions of the plants and the distillation process. Environmental factors, harvesting timing, and post-processing practices can alter the chemotype of essential oils [41]. Moreover, the distillation process itself can affect the composition of essential oils; for instance, the timing of collection during distillation can influence the chemical profile and aroma. In this study, we controlled several variables, including geographical location, tree age, post-processing of *C. kanehirai* Hayata, heartwood-to-water ratio, and the timing of essential oil collection during distillation. However, the potential effects of environmental factors (e.g., temperature) and harvesting conditions (e.g., season) on the composition and anti-inflammatory activity of the EOC require further investigation. Another limitation of this study is the absence of animal studies to confirm the inhibitory activity of EOC on the NLRP3 inflammasome and inflammation in vivo. The mouse models for NLRP3 inflammasome-associated inflammatory diseases used in our previous studies—including DSS-induced colitis [42], uric acid crystal-induced peritonitis [43], and IgA immune complex-induced IgA nephropathy [44]—can be employed in future research to further explore in vivo efficacy and safety. Additionally, one of the key criteria for candidate molecules in new drug development is favorable pharmacokinetic properties, including absorption, distribution, metabolism, and excretion (ADME). Although some plant essential oils have demonstrated good bioaccessibility and bioavailability in vivo [45,46], further studies are necessary to determine the pharmacokinetic profile of EOC.

## 4. Materials and Methods

### 4.1. Reagents

LPS from *Escherichia coli* O111:B4 and phorbol 12-myristate 13-acetate (PMA) were obtained from Sigma-Aldrich (St. Louis, MO, USA). ATP, MSU crystals, CPPD crystals, Alum, Nano-SiO_2_, MDP, FLA-ST, poly(dA/dT), Pam3CSK4, NF-κB-inducible secreted alkaline phosphatase (SEAP) reporter plasmid with Zeocin resistance gene (pNiFty2-N-SEAP-Zeo), QUANTI-Blue solution, and Zeocin were sourced from InvivoGen (San Diego, CA, USA). The AlamarBlue assay kit was purchased from AbD Serotec (Oxford, UK). Mitotracker Deep Red, Mitotracker Green, MitoSOX, and CM-H_2_DCFDA were acquired from Thermo Fisher Scientific (Waltham, MA, USA). ELISA kits for IL-1β, IL-6, and TNF-α were sourced from R&D Systems (Minneapolis, MN, USA). An ELISA kit for caspase-1 was purchased from IBL-America (Minneapolis, MN, USA). Antibodies against NLRP3 and caspase-1 were obtained from Adipogen International (San Diego, CA, USA). Antibodies against ASC and actin were acquired from Santa Cruz Biotechnology (Santa Cruz, CA, USA).

### 4.2. Plant Materials

The heartwood of *C. kanehirai* Hayata was collected from the Taimali Research Center of the Taiwan Forestry Research Institute (coordinates: 22.59942, 120.98011). The species was identified by Chen-Lung Ho of the Division of Wood Cellulose at the Taiwan Forestry Research Institute in Taipei, Taiwan.

### 4.3. Isolation of EOC

Essential oil was extracted from the heartwood of *C. kanehirai* Hayata by hydrodistillation using a Clevenger apparatus, following the method described in our previous study with minor modifications [47]. For each extraction, 5 kg of heartwood was distilled for 3 h, and the procedure was repeated three times. The resulting essential oil was dried over anhydrous sodium sulfate to remove residual moisture, then transferred to individual sample vials and stored at 4 °C to maintain stability and quality for subsequent analysis and applications. The essential oil yield and related experimental data were based on three replicates, and results are expressed as the mean ± standard deviation.

### 4.4. EOC Analysis

To analyze the composition of EOC, gas chromatography–flame ionization detection (GC–FID) and GC–MS were employed, following the method described in our previous study with minor modifications [47]. GC–FID analysis was conducted using a Hewlett–Packard Model 6890 gas chromatograph (Agilent Technologies, Inc., Santa Clara, California, USA) equipped with a flame ionization detector (FID). The system was fitted with a DB-5 fused silica capillary column (5% phenyl, 95% methylpolysiloxane), 30 m in length, with an internal diameter of 0.25 mm and a film thickness of 0.25 μm. The oven temperature was initially set at 50 °C and held for 2 min, then increased at a rate of 5 °C/min to 250 °C. The injector and detector temperatures were maintained at 270 °C and 250 °C, respectively. Hydrogen was used as the carrier gas at a flow rate of 1.0 mL/min, with a split ratio of 1:60. Prior to injection, 1 μL of essential oil was diluted 1:100 (*v/v*) with ethyl acetate. Linear retention indices (LRIs) of the compounds were determined by comparing their retention times with those of a C8-C30 n-alkane standard series. The relative content of each component was quantified by electronic integration of peak areas using the normalization method, without applying correction factors. GC–MS analysis was carried out under the same chromatographic conditions using a Hewlett–Packard Model 6890/5973 GC–MS system (Agilent Technologies, Inc., Santa Clara, California, USA) with the same DB-5 column. High-purity helium (99.995%) was used as the carrier gas at a flow rate of 1.0 mL/min. The data were acquired in full scan mode over a mass-to-charge ratio (m/z) range of 30–500.

### 4.5. Component Identification

The compounds were identified by comparing their LRIs, retention times, and mass spectra with data from the NIST and Wiley mass spectral libraries, authentic reference standards, and relevant literature sources [20,21,22].

### 4.6. Cell Cultures

The mouse J774A.1 macrophage cell line and human THP-1 monocytic cell line were obtained from the Bioresource Collection and Research Center at the Food Industry Research and Development Institute in Hsinchu, Taiwan. To generate THP-1 macrophages, THP-1 monocytic cells were differentiated into macrophages by culturing them for 48 h in RPMI-1640 medium supplemented with 100 nM PMA. To generate NF-κB reporter cells, J774A.1 macrophages were stably transfected with NF-κB-inducible SEAP reporter plasmids and subsequently selecting them using 100 μg/mL of Zeocin. All cells were maintained in RPMI-1640 medium containing 10% heat-inactivated fetal bovine serum at 37 °C in a 5% CO_2_ incubator.

### 4.7. Effect of EOC on the Activation of Inflammasomes

To investigate the effect of EOC on NLRP3 inflammasome activation, we employed two models. In Model 1, J774A.1 macrophages were incubated with EOC or vehicle (0.1% DMSO) for 0.5 h, followed by priming with 1 µg/mL LPS for 5.5 h. The cells were then activated with 5 mM ATP for 0.5 h. In Model 2, either J774A.1 macrophages or THP-1 macrophages were first primed with 1 µg/mL LPS for 5.5 h, then incubated with EOC or vehicle for 0.5 h. The cells were subsequently activated with either 5 mM ATP, 10 µM nigericin for 0.5 h, or with 100 µg/mL MSU crystals, 100 µg/mL nano-SiO_2_, 10 µg/mL CPPD crystals, or 100 µg/mL Alum for 24 h. In both Model 1 and Model 2, the concentration of IL-1β and caspase-1 in the supernatants was analyzed using ELISA. To examine the effect of EOC on inflammasome activation beyond the NLRP3 inflammasome, J774A.1 macrophages were primed with 1 µg/mL LPS or 1 µg/mL Pam3CSK4 for 5.5 h, followed by incubation with EOC or vehicle for 0.5 h. The cells were then activated by transfection with 10 µg/mL MDP (NLRP1 inflammasome), 1 µg/mL FLA-ST (NLRC4 inflammasome), 2 µg/mL poly(dA/dT) (AIM2 inflammasome), or 2 µg/mL LPS (non-canonical inflammasome) for 6 h. The concentration of IL-1β in the supernatants was analyzed using ELISA.

### 4.8. Effect of EOC on Pro-Inflammatory Mediator Expression in LPS-Activated Macrophages

To assess the effect of EOC on the expression of TNF-α, IL-6, and the production of NO, J774A.1 macrophages were incubated with EOC for 0.5 h, followed by activation with 1 µg/mL LPS for 24 h. The concentrations of TNF-α and IL-6 in the supernatants were measured by ELISA, while the NO concentration in the supernatants was determined using the Griess reaction.

### 4.9. Effect of EOC on the ROS Production

J774A.1 macrophages were incubated with EOC or vehicle for 30 min, followed by activation with 1 µg/mL LPS for durations ranging from 5 to 80 min. The cells were then stained with 2 µM CM-H_2_DCFDA for 15 min, and the production of intracellular ROS was assessed by measuring fluorescent signals using a spectrophotometer (BMG SPECTROstar Nano, Ortenberg, Germany), with an excitation wavelength of 485 nm and an emission wavelength of 530 nm.

### 4.10. Effect of EOC on the Transcriptional Activity of NF-κB

NF-κB reporter cells were incubated with either 100 µg/mL EOC or the vehicle for 30 min, followed by activation with 1 µg/mL LPS for 24 h. After incubation, 180 μL of QUANTI-Blue solution was added to each well of a 96-well plate, mixed with 20 μL of culture supernatant from each group. The plate was then incubated at 37 °C for 30 min, and the optical density was measured at 620 nm using a spectrophotometer. The transcriptional activity of NF-κB is expressed as the fold change in optical density compared to the control group.

### 4.11. Effect of EOC on the Mitochondrial Damage

J774A.1 macrophages were primed with 1 µg/mL LPS for 5.5 h and then incubated with either 100 μg/mL EOC or the vehicle for 0.5 h. Following this, the cells were activated with 5 mM ATP or 10 μM nigericin for 0.5 h. The cells were stained with 5 nM MitoSOX to detect mitochondrial ROS, or with a combination of 25 nM MitoTracker Deep Red and 25 nM MitoTracker Green to assess mitochondrial membrane integrity, for 15 min. The fluorescence signals were subsequently analyzed using flow cytometry (Cytomics FC500 Flow Cytometry CXP, Beckman Coulter Life Sciences, Indianapolis, IN, USA).

### 4.12. Effect of EOC on the Cell Viability

We seeded 1000 J774A.1 macrophages in each well of a 96-well plate using 100 μL of culture medium per well and incubated them overnight in a cell incubator. The J774A.1 macrophages were treated with EOC at concentrations ranging from 50 to 800 µg/mL or with the vehicle (serving as the negative control) for 24 h, with or without the addition of 1 µg/mL LPS. Wells containing culture medium without cells were included as background controls. After treatment, we added 10 μL of Alamar Blue solution to each well and incubated it at 37 °C, protected from light, for 2 h. The signals were measured using a spectrophotometer with an excitation wavelength of 570 nm and an emission wavelength of 595 nm. Cell viability was calculated using the following formula: Cell viability (%) = 100 × (fluorescence intensity of the experimental group − fluorescence intensity of the background control)/(fluorescence intensity of the negative control − fluorescence intensity of the background control).

### 4.13. Statistical Analysis

GraphPad Prism version 6.01 was used for graphing, while IBM SPSS Statistics version 24 was employed for statistical analysis. Two-tailed *t*-tests were conducted for comparisons between two groups, and ANOVA followed by Dunnett’s multiple comparisons test was applied for comparisons involving three or more groups. We set the alpha level at 0.05 to determine statistical significance, a widely accepted threshold in scientific research to minimize the risk of Type I errors. Error bars in the figures represent the standard deviation calculated from three independent experiments. Asterisks indicate significance levels, with *, **, and *** corresponding to *p*-values of <0.05, <0.01, and <0.001, respectively.

## 5. Conclusions

Our current study highlights the significant anti-inflammatory and anti-NLRP3 inflammasome activities of EOC in macrophages. Terpinen-4-ol, the primary compound in EOC, is likely responsible for its anti-inflammatory effects. Since uncontrolled inflammation and aberrant activation of the NLRP3 inflammasome are associated with the development of inflammatory diseases linked to obesity, aging, and infections, EOC shows promise as a natural agent for promoting health and preventing such conditions in the future.

## Figures and Tables

**Table 1:** Chemical composition of EOC.

Peak No.	Compound I.D.	Classification ^a^	LRILit ^b^	LRIExp ^c^	Concentration (%)	Identification ^d^
1	α-Pinene	MH	939	938	0.2	MS,LRI,CO-ST
2	β-Myrcene	MH	991	992	0.5	MS,LRI,CO-ST
3	α-Phellandrene	MH	1003	1005	0.3	MS,LRI,CO-ST
4	1,4-Cineole	OM	1015	1015	11.9	MS,LRI,CO-ST
5	*p*-Cymene	MH	1025	1024	9.3	MS,LRI,CO-ST
6	Limonene	MH	1029	1029	3.3	MS,LRI,CO-ST
7	1,8-Cineole	OM	1031	1033	0.5	MS,LRI,CO-ST
8	γ-Terpinene	MH	1060	1059	9.7	MS,LRI,CO-ST
9	Terpinolene	MH	1089	1089	1.9	MS,LRI,CO-ST
10	*p*-Cymenene	MH	1091	1093	0.2	MS,LRI,CO-ST
11	1-Terpineol	OM	1134	1135	2.4	MS,LRI
12	*trans-p*-Menthan-8-ol	OM	1148	1149	0.4	MS,LRI
13	Terpinen-4-ol	OM	1177	1178	20.5	MS,LRI,CO-ST
14	α-Terpineol	OM	1189	1191	1.0	MS,LRI,CO-ST
15	γ-Terpineol	OM	1199	1199	0.2	MS,LRI,CO-ST
16	*m*-Propylphenol	OM	1236	1236	0.3	MS,LRI
17	Safrole	OM	1287	1288	34.5	MS,LRI,CO-ST
18	α-Terpinyl acetate	OM	1349	1351	0.4	MS,LRI,CO-ST
19	(*E*)-Isosafrole	OM	1376	1374	0.4	MS,LRI
20	(*E*)-β-Farnesene	SH	1457	1458	1.0	MS,LRI
21	δ-Cadinene	SH	1523	1526	0.7	MS,LRI
22	t-Muurolol	OS	1642	1643	0.3	MS,LRI
23	α-Cadinol	OS	1654	1656	0.2	MS,LRI
Monoterpene hydrocarbons (%)					25.4	
Oxygenated monoterpenes (%)					72.6	
Sesquiterpene hydrocarbons (%)					1.7	
Oxygenated sesquiterpenes (%)					0.4	
Oil Yield (mL/100 g)					2.58 ± 0.02	

^a^ Classification: MH = Monoterpene hydrocarbons; OM = Oxygenated monoterpenes; SH = Sesquiterpene hydrocarbons; OS = Oxygenated sesquiterpenes. ^b^ LRILit = LRI values from a previous study [20]. ^c^ LRIExp = computed LRI values obtained for a mixture of a continuous series of n-alkane hydrocarbons (C8 to C30) in a DB-5 capillary column. ^d^ Identification through MS = comparison of the NIST and Wiley mass spectral libraries; LRI = linear retention index; (LRI) same as the previous findings [20,21,22]; and CO-ST = co-injection/comparison with the LRI and MS standards.

## Data Availability

Data will be made available on request.

## Abbreviations

The following abbreviations are used in this manuscript:

EOC	Essential oil derived from the heartwood of *Cinnamomum kanehirai* Hayata
IL	Interleukin
TNF-α	Tumor necrosis factor-α
NLRP3	NLR family pyrin domain containing 3
ASC	Apoptosis-associated speck-like protein
ROS	Reactive oxygen species
LPS	Lipopolysaccharide
ATP	Adenosine triphosphate
MSU	Monosodium urate
CPPD	Calcium pyrophosphate dihydrate
Alum	Aluminum hydroxide
Nano-SiO_2_	SiO_2_ nanoparticles
MDP	Muramyl dipeptide
FLA-ST	Flagellin from *Salmonella typhimurium*
Pam3CSK4	Synthetic triacylated lipopeptide
GC–MS	Gas chromatography–mass spectrometry

## References

[1] Muralidharan S., Mandrekar P. (2013). Cellular stress response and innate immune signaling: Integrating pathways in host defense and inflammation. J. Leukoc. Biol..

[2] Koh T.J., DiPietro L.A. (2011). Inflammation and wound healing: The role of the macrophage. Expert Rev. Mol. Med..

[3] Ritter B., Greten F.R. (2019). Modulating inflammation for cancer therapy. J. Exp. Med..

[4] Glass C.K., Saijo K., Winner B., Marchetto M.C., Gage F.H. (2010). Mechanisms underlying inflammation in neurodegeneration. Cell.

[5] Fulop T., Larbi A., Pawelec G., Khalil A., Cohen A.A., Hirokawa K., Witkowski J.M., Franceschi C. (2023). Immunology of Aging: The Birth of Inflammaging. Clin. Rev. Allergy Immunol..

[6] Schleh M.W., Caslin H.L., Garcia J.N., Mashayekhi M., Srivastava G., Bradley A.B., Hasty A.H. (2023). Metaflammation in obesity and its therapeutic targeting. Sci. Transl. Med..

[7] Fu J., Wu H. (2023). Structural Mechanisms of NLRP3 Inflammasome Assembly and Activation. Annu. Rev. Immunol..

[8] Latz E., Duewell P. (2018). NLRP3 inflammasome activation in inflammaging. Semin. Immunol..

[9] Wang H., Ma L., Su W., Liu Y., Xie N., Liu J. (2025). NLRP3 inflammasome in health and disease (Review). Int. J. Mol. Med..

[10] Ma Q. (2023). Pharmacological Inhibition of the NLRP3 Inflammasome: Structure, Molecular Activation, and Inhibitor-NLRP3 Interaction. Pharmacol. Rev..

[11] Kelley N., Jeltema D., Duan Y., He Y. (2019). The NLRP3 Inflammasome: An Overview of Mechanisms of Activation and Regulation. Int. J. Mol. Sci..

[12] Liu Q., Zhang D., Hu D., Zhou X., Zhou Y. (2018). The role of mitochondria in NLRP3 inflammasome activation. Mol. Immunol..

[13] Bagherniya M., Khedmatgozar H., Fakheran O., Xu S., Johnston T.P., Sahebkar A. (2021). Medicinal plants and bioactive natural products as inhibitors of NLRP3 inflammasome. Phytother. Res..

[14] Jahan S., Kumar D., Chaturvedi S., Rashid M., Wahajuddin M., Khan Y.A., Goyal S.N., Patil C.R., Mohanraj R., Subramanya S. (2017). Therapeutic Targeting of NLRP3 Inflammasomes by Natural Products and Pharmaceuticals: A Novel Mechanistic Approach for Inflammatory Diseases. Curr. Med. Chem..

[15] Lee M.H., Jiang C.B., Juan S.H., Lin R.D., Hou W.C. (2006). Antioxidant and heme oxygenase-1 (HO-1)-induced effects of selected Taiwanese plants. Fitoterapia.

[16] Liu Y.K., Chen K.H., Leu Y.L., Way T.D., Wang L.W., Chen Y.J., Liu Y.M. (2014). Ethanol extracts of *Cinnamomum kanehirai* Hayata leaves induce apoptosis in human hepatoma cell through caspase-3 cascade. Onco Targets Ther..

[17] Yeh R.Y., Shiu Y.L., Shei S.C., Cheng S.C., Huang S.Y., Lin J.C., Liu C.H. (2009). Evaluation of the antibacterial activity of leaf and twig extracts of stout camphor tree, *Cinnamomum kanehirae*, and the effects on immunity and disease resistance of white shrimp, *Litopenaeus vannamei*. Fish Shellfish Immunol..

[18] Lundberg J.O., Weitzberg E. (2022). Nitric oxide signaling in health and disease. Cell.

[19] Facchin B.M., Dos Reis G.O., Vieira G.N., Mohr E.T.B., da Rosa J.S., Kretzer I.F., Demarchi I.G., Dalmarco E.M. (2022). Inflammatory biomarkers on an LPS-induced RAW 264.7 cell model: A systematic review and meta-analysis. Inflamm. Res..

[20] Adams R.P. (2007). Identification of Essential Oil Components by Gas Chromatography/Mass Spectrometry.

[21] Van den Dool H., Kratz P.D. (1963). A generalization of the retention index system including linear temperature programmed gas liquid partition chromatography. J Chromatogr. A.

[22] NIST NIST Chemistry Web Book: NIST Standard Reference Database Number 69. http://webbook.nist.gov/chemistry/.

[23] Miyah Y., Benjelloun M., Lairini S., Lahrichi A. (2022). COVID-19 Impact on Public Health, Environment, Human Psychology, Global Socioeconomy, and Education. Sci. World J..

[24] Rodrigues T.S., de Sá K.S.G., Ishimoto A.Y., Becerra A., Oliveira S., Almeida L., Gonçalves A.V., Perucello D.B., Andrade W.A., Castro R. (2021). Inflammasomes are activated in response to SARS-CoV-2 infection and are associated with COVID-19 severity in patients. J. Exp. Med..

[25] Bonaventura A., Vecchié A., Dagna L., Tangianu F., Abbate A., Dentali F. (2022). Colchicine for COVID-19: Targeting NLRP3 inflammasome to blunt hyperinflammation. Inflamm Res..

[26] Saeedi-Boroujeni A., Mahmoudian-Sani M.R., Nashibi R., Houshmandfar S., Tahmaseby Gandomkari S., Khodadadi A. (2021). Tranilast: A potential anti-Inflammatory and NLRP3 inflammasome inhibitor drug for COVID-19. Immunopharmacol. Immunotoxicol..

[27] Li L.H., Chiu H.W., Wong W.T., Huang K.C., Lin T.W., Chen S.T., Hua K.F. (2023). *Antrodia cinnamomea* May Interfere with the Interaction Between ACE2 and SARS-CoV-2 Spike Protein in vitro and Reduces Lung Inflammation in a Hamster Model of COVID-19. J. Inflamm. Res..

[28] Galozzi P., Bindoli S., Luisetto R., Sfriso P., Ramonda R., Scanu A., Oliviero F. (2021). Regulation of crystal induced inflammation: Current understandings and clinical implications. Expert Rev. Clin. Immunol..

[29] Martinon F., Pétrilli V., Mayor A., Tardivel A., Tschopp J. (2006). Gout-associated uric acid crystals activate the NALP3 inflammasome. Nature.

[30] Jia H., Liu Y., Guo D., He W., Zhao L., Xia S. (2021). PM2.5-induced pulmonary inflammation via activating of the NLRP3/caspase-1 signaling pathway. Environ. Toxicol..

[31] Rathinam V.A., Jiang Z., Waggoner S.N., Sharma S., Cole L.E., Waggoner L., Vanaja S.K., Monks B.G., Ganesan S., Latz E. (2010). The AIM2 inflammasome is essential for host defense against cytosolic bacteria and DNA viruses. Nat. Immunol..

[32] Paulin N., Viola J.R., Maas S.L., de Jong R., Fernandes-Alnemri T., Weber C., Drechsler M., Döring Y., Soehnlein O. (2018). Double-Strand DNA Sensing Aim2 Inflammasome Regulates Atherosclerotic Plaque Vulnerability. Circulation.

[33] Zhang L., Chen S., Ruan J., Wu J., Tong A.B., Yin Q., Li Y., David L., Lu A., Wang W.L. (2015). Cryo-EM structure of the activated NAIP2-NLRC4 inflammasome reveals nucleated polymerization. Science.

[34] de Sousa D.P., Damasceno R.O.S., Amorati R., Elshabrawy H.A., de Castro R.D., Bezerra D.P., Nunes V.R.V., Gomes R.C., Lima T.C. (2023). Essential Oils: Chemistry and Pharmacological Activities. Biomolecules.

[35] Hua K.F., Yang T.J., Chiu H.W., Ho C.L. (2014). Essential oil from leaves of *Liquidambar formosana* ameliorates inflammatory response in lipopolysaccharide-activated mouse macrophages. Nat. Prod. Commun..

[36] Zhang Z., Shen P., Lu X., Li Y., Liu J., Liu B., Fu Y., Cao Y., Zhang N. (2017). In Vivo and In Vitro Study on the Efficacy of Terpinen-4-ol in Dextran Sulfate Sodium-Induced Mice Experimental Colitis. Front. Immunol..

[37] Pelegrin P., Barroso-Gutierrez C., Surprenant A. (2008). P2X7 receptor differentially couples to distinct release pathways for IL-1beta in mouse macrophage. J. Immunol..

[38] de Oliveira M.G., Marques R.B., de Santana M.F., Santos A.B., Brito F.A., Barreto E.O., De Sousa D.P., Almeida F.R., Badauê-Passos D., Antoniolli A.R. (2012). α-terpineol reduces mechanical hypernociception and inflammatory response. Basic Clin. Pharmacol. Toxicol..

[39] Ma S., Yang B., Du Y., Lv Y., Liu J., Shi Y., Huang T., Xu H., Deng L., Chen X. (2023). 1,8-cineole ameliorates colon injury by downregulating macrophage M1 polarization via inhibiting the HSP90-NLRP3-SGT1 complex. J. Pharm. Anal..

[40] Zhang J., Li X., Cui W., Lu D., Zhang Y., Liao J., Guo L., Jiao C., Tao L., Xu Y. (2024). 1,8-cineole ameliorates experimental diabetic angiopathy by inhibiting NLRP3 inflammasome-mediated pyroptosis in HUVECs via SIRT2. Biomed. Pharmacother..

[41] Belabbes R., Dib M.E.A., Djabou N., Ilias F., Tabti B., Costa J., Muselli A. (2017). Chemical Variability, Antioxidant and Antifungal Activities of Essential Oils and Hydrosol Extract of *Calendula arvensis* L. from Western Algeria. Chem. Biodivers..

[42] Chiu H.W., Wu C.H., Lin W.Y., Wong W.T., Tsai W.C., Hsu H.T., Ho C.L., Cheng S.M., Cheng C.C., Yang S.P. (2024). The Angiotensin II Receptor Neprilysin Inhibitor LCZ696 Inhibits the NLRP3 Inflammasome By Reducing Mitochondrial Dysfunction in Macrophages and Alleviates Dextran Sulfate Sodium-induced Colitis in a Mouse Model. Inflammation.

[43] Hsieh C.Y., Li L.H., Rao Y.K., Ju T.C., Nai Y.S., Chen Y.W., Hua K.F. (2019). Mechanistic insight into the attenuation of gouty inflammation by Taiwanese green propolis via inhibition of the NLRP3 inflammasome. J. Cell. Physiol..

[44] Tsai Y.L., Hua K.F., Chen A., Wei C.W., Chen W.S., Wu C.Y., Chu C.L., Yu Y.L., Lo C.W., Ka S.M. (2017). NLRP3 inflammasome: Pathogenic role and potential therapeutic target for IgA nephropathy. Sci. Rep..

[45] Marques R.B., Barreto Sousa M.D.D., de Sousa Santos W., de Barros Leite N.F., Sobreiro Lima E.M., Lima Soares A., da Costa C.L.S., Silva Filho F.A.e., Maia Filho A.L.M., Soares Martins E.P. (2023). Pharmacokinetic and toxicological prediction of the chemical constituents of the essential oil of the leaves of Croton heliotropiifolius Kunth. J. Toxicol. Environ. Health A.

[46] Hou M.Z., Chen L.L., Chang C., Zan J.F., Du S.M. (2021). Pharmacokinetic and tissue distribution study of eight volatile constituents in rats orally administrated with the essential oil of *Artemisiae argyi* Folium by GC-MS/MS. J. Chromatogr. B.

[47] Ho C.L., Li L.H., Weng Y.C., Hua K.F., Ju T.C. (2020). Eucalyptus essential oils inhibit the lipopolysaccharide-induced inflammatory response in RAW264.7 macrophages through reducing MAPK and NF-κB pathways. BMC Complement. Med. Ther..

